# The impact of a risk assessment tool on hospital pressure injury prevalence and prevention: a quantitative pre-post evaluation

**DOI:** 10.1016/j.ijnsa.2025.100342

**Published:** 2025-05-01

**Authors:** Ragnar Seton, Elisabeth Wetzer, Lisa Hultin

**Affiliations:** a Department of Physics and Technology, UiT The Arctic University of Norway, Tromsø, Norway; b Department of Public Health and Caring Sciences, Uppsala University, Uppsala, Sweden; c Uppsala university hospital, Sweden

**Keywords:** Evaluation study, Pressure injury/pressure ulcer, Nursing, Prevention, Risk assessment, Quantitative

## Abstract

**Background:**

Pressure injuries are preventable adverse events, and their prevalence is established as a quality indicator in healthcare. They are a notable healthcare problem worldwide, as they have a significant impact on the quality of life of affected individuals and are associated with high costs for healthcare systems. The use of an evidence-based risk assessment instrument is, therefore, crucial to enable efficient, effective, and reliable assessments.

**Objective:**

The evidence-based pressure injury risk assessment instrument PURPOSE T was widely introduced at a university hospital in 2021, replacing another commonly used assessment tool, the Modified Norton Scale. In this study, we quantified the prevalence of pressure injuries before and after the introduction of PURPOSE T to measure the tool’s impact.

**Settings:**

At a university hospital in Sweden with about 900 beds.

**Methods:**

We adopted a cross-sectional research design and analysed records from point prevalence measurements carried out between September 2018 and 2023. The number of patients included in each point prevalence measurement varied between 474 and 633. Prevalence of all pressure injuries and prescribed prevention interventions over a 5-year period was calculated, compared, and analysed.

**Results:**

We observed notable increases in the use of preventive interventions, particularly pressure-reducing chair cushions (+28.6 %) and position adjustments in chairs (+24.2 %). Additionally, we found an average increase of +23.0 % in the prevalence of category I pressure injuries after the introduction of PURPOSE T while observing a decrease in most severe pressure injury categories:35.5 % in category III and -35.3 % in category IV.

**Conclusions:**

We found that the introduction of PURPOSE T contributed to the increased use of preventive interventions. This, in turn, was associated with a decrease or modification in the prevalence of pressure injuries, suggesting a positive impact on pressure injury within this facility.

**What is already known**

• Pressure injuries are preventable adverse events and are recognized as a key quality indicator in healthcare.
• There is a knowledge gap regarding the impact of evidence-based pressure injury risk assessment instruments and their validity.
• Over 40 risk assessment instruments for pressure injuries, many developed in the 1960s and still in use today, rely on outdated medical knowledge unsupported by state-of-the-art insight.

Alt-text: Unlabelled box

**What this paper adds**

• We described the impact of the implementation of a new evidence-based risk assessment instrument for pressure injuries at a university hospital in Sweden, based on surveys over a 5-year period.
• Within the 5-year period, the prevalence of pressure injuries was measured; Category I (least severe) increased and Categories III and IV (most severe) decreased by >35 %.
• Despite the successful widespread implementation that engaged hospital staff in risk assessing patients and preventing pressure injuries, one in every 30 patients still suffered from category III or IV pressure injuries.

Alt-text: Unlabelled box

## 1 Introduction

Pressure injuries, also known as pressure ulcers, are a significant concern in healthcare, impacting patient outcomes and healthcare costs worldwide (Gorecki et al., 2012; Demarré et al., 2015; European Pressure Ulcer Advisory Panel (EPUAP), 2019). Despite advances in preventive strategies and the development of several risk assessment tools, pressure injuries remain prevalent, posing a persistent challenge in clinical settings (EPUAP, 2019). We aimed to explore the efficacy of the recently implemented Pressure Ulcer Primary or Secondary Evaluation Tool (PURPOSE T) (Coleman et al., 2018; Hultin et al., 2021) at a major Swedish university hospital. By evaluating the impact of PURPOSE T, we sought to contribute to the ongoing efforts to enhance pressure injury prevention and improve patient care.

## 2 Background

Pressure injuries are preventable adverse events; as such, they are recognized as a key quality indicator in healthcare (EPUAP, 2019). Individuals who suffer from pressure injuries often experience a significant decline in health-related quality of life, as these injuries impose a substantial burden on patients (Gorecki et al., 2012). Consequently, it is imperative for the healthcare system to address risk factors that contribute to the prevalence of pressure injuries across the continuum of care, especially as pressure injuries are also associated with considerable healthcare costs (Demarré et al., 2015). Both international and Swedish guidelines exist to aid in the prevention of pressure injuries. According to these guidelines, prevention begins with identifying patients at risk of developing pressure injuries through comprehensive risk assessment, which should be conducted as soon as possible after admission (EPUAP, 2019). These guidelines agree that a structured pressure injury risk assessment instrument is fundamental to pressure injury prevention. However, pressure injury risk assessment and prevention are complex interventions (EPUAP, 2019). Since the early 1960s, over 40 pressure injury risk assessment instruments have been developed (Huang et al. 2021). Coleman et al. (2013) conducted a systematic review that found that these instruments had limitations in their methodological development, with no significant differences in their ability to identify patients at risk for pressure injuries. It has been suggested that pressure injury risk assessment instruments should be developed based on a multivariable analysis to identify factors independently associated with pressure injury development (Nixon and McGough, 2001; EPUAP, 2019).

Internationally, the Braden Scale (Braden and Bergstrom, 1987) is one of the most used pressure injury risk assessment instruments, while in Sweden the Modified Norton Scale (Ek, 1987) is the most widely used (Källman and Lindgren, 2014). Both instruments require healthcare staff to perform a full assessment of all patients, even those who clearly appear not to be at risk, consuming valuable and already limited nursing time (Coleman et al., 2018; Hultin et al., 2021).

The Modified Norton Scale evaluates seven risk factors: mental condition, physical activity, mobility, food intake, fluid intake, incontinence, and general physical condition. For many years, it has been standard practice to use numeric scales that generate a total score, which is then compared to a reference value to determine whether the patient is *at risk* or *not at risk* (Källman and Lindgren, 2014). However, the Modified Norton Scale does not include all risk factors deemed important by the latest evidence in international guidelines, such as skin status and circulation (Coleman et al., 2013; EPUAP, 2019). Hultin et al. (2020, 2022) indicated that the Modified Norton Scale is considered outdated by registered nurses (RNs), who report that it sometimes fails to identify patients at risk, while their clinical judgement indicates that the patient is at risk or, even worse, when the patient already suffers from a pressure injury.

In response to the need for a new evidence-based pressure injury risk assessment instrument, Coleman et al. (2018) developed PURPOSE T. The development of this instrument involved a systematic review of pressure injury risk factors, a consensus study with service user involvement, conceptual framework development, pre-testing with clinical nurses, and clinical evaluation in both acute and community settings (Coleman et al., 2013,^2014^,^2016^;^2018^). The instrument considers risk factors recommended by the most current evidence, including skin assessment. It differs from traditional risk assessment instruments in that it identifies both patients at risk of developing pressure injuries and those who already have existing pressure injuries. The tool consists of three steps, culminating in a reflective assessment of the patient's risk. The process also provides a natural opportunity to engage with the patient and inform them about the result of the risk assessment.

PURPOSE T’s three-step assessment proceeds as follows (in the instrument, the term pressure ulcers is used, which is synonymous with pressure injury). *(1)* A screening assessment that includes mobility, skin status, and clinical judgement, which allows for patients who are clearly not at risk to be quickly screened out. *(2)* A full assessment that evaluates independent movement, sensory perception, moisture, diabetes, circulation, nutrition, medical devices, detailed skin assessment, and previous pressure injuries. *(3)* Based on the results of Step 2, one of three assessment decisions is made: *no pressure ulcer - not currently at risk, no pressure ulcer - but at risk*, or *pressure ulcer or a scar from pressure ulcer* (Coleman, 2016). It provides an approach to pressure injury risk assessment incorporating a screening stage to allow those who are clearly not at risk to be quickly cleared and a full assessment stage, facilitating a comprehensive assessment for those potentially at risk, while encouraging users to use their clinical judgement as part of the assessment process. It encourages a more holistic and tailored approach for care planning, promoting consideration of the individual patient’s risk profile, rather than a numerical score as used in traditional pressure injury risk assessment instruments.

PURPOSE T was translated into Swedish in 2019 and evaluated in a clinical context, demonstrating good psychometric properties, usability and, feasibility in Sweden (Hultin et al., 2020, 2021, 2022). Following its evaluation, it was widely implemented across a university hospital; all units (with the exception of the children’s ward) were included in the spring of 2021.

In theory, the use of PURPOSE T could lead to the instigation of more appropriate preventative interventions and individualized care planning, that in turn, would enable improved care or pressure injury outcomes. However, existing evidence about traditional pressure injury risk assessment instruments suggests poor linkage between the assessment outcome and the selection of preventative interventions and pressure injury incidence. It is, therefore, essential to evaluate the implementation of PURPOSE T to understand its impact on patient outcome. To date, no evaluations have been conducted on the widespread implementation of this instrument.

## 3 Objectives

The aim of this study was to evaluate the impact of the implementation of PURPOSE T at a university hospital in Sweden.

The driving questions of this study were as follows:

1. How were pressure injury preventive measures implemented before and after the implementation of PURPOSE T?
2. How was the prevalence of pressure injuries affected by the implementation of PURPOSE T?

## 4 Method

### 4.1 Study design

A cross-sectional research design was adopted.

Between September of 2018 and 2023, the data collection of all point prevalence measurements of prescribed prevention and pressure injuries at the hospital in the study was kept in a fixed format, thus minimizing ambiguity in the records and ensuring that comparisons of aggregated records could be performed over the time span. With PURPOSE T introduced at the hospital in May 2021, this dataset was used to quantify the changes in prescribed prevention interventions and compare the pressure injury prevalence before and after the introduction.

### 4.2 Setting/participants

We conducted the study at a Swedish university hospital with approximately 900 beds. Most nurses in Sweden hold a bachelor of science degree in nursing, while assistant nurses have a certification from upper secondary school. RNs are primarily responsible for pressure injury risk assessments, care planning, and documentation in electronic health records. They also work in teams, together with assistant nurses. The university hospital in the study adheres to the national guidelines from the Swedish Association of Local Authorities and Regions, which are based on international guidelines (EPUAP, 2019). All patients admitted to the hospital should undergo a skin assessment, and those who are bedridden, in a wheelchair, have low mobility, have sensitive or fragile skin, or already have a pressure injury should be risk assessed as soon as possible following admission. The assessment should be repeated regularly, particularly after major surgery or a decline in health condition or if the patient is transferred to another ward. All patients identified as *at risk* should receive preventive nursing interventions.

At the hospital, point prevalence measurements of prescribed prevention and pressure injuries have been conducted three times per year since 2018, following a measurement at the beginning of that year that revealed a pressure injury prevalence of 25 % (according to EPUAP methodology, 2019). This increased measurement frequency was implemented in response to the high prevalence rate (the dates were customized to the availability of staff; therefore, no point prevalence measurements were collected during the summer months).

### 4.3 Data collection/procedure

The pseudonymized data were collected from a database at the hospital and consisted of the records from point prevalence measurements during which all inpatients were assessed for their pressure injury risk. The records used in this study spanned from 2018 to 2023 (5-year period). Between 2018 and 2021, guidelines of the hospital were to risk assess using the Modified Norton Scale; since 2021, the guidelines were to use PURPOSE T; i.e. the years 2021–2023 were based on PURPOSE T.

The methodology used was developed by the European Pressure Ulcer Advisory Panel (EPUAP, 2019) and was followed during all point prevalence measurements. The Department of Quality and Patient Safety at the hospital choses the dates and notifies all wards. At each ward, a team of RNs or assistant nurses (trained by local supervisors from the Department of Quality and Patient Safety) was responsible for data collection at each point prevalence measurement. All protocols used in the data collection over the 5-year period included patient data, such as sex, age, skin assessment, preventive intervention, and risk assessment. The preventive interventions included, for example, the type of mattress, the use of seat cushions, and repositioning in bed or seating. All patients 18 years and older were asked for consent to be risk assessed. Pressure injuries were identified by the visual skin assessment, and any existing pressure injuries were categorized according to the European Pressure Ulcer Advisory Panel classification system.

We chose to focus the scope of this study on the direct effects of the instrument, as the impact of a widespread implementation of a pressure injury risk assessment instrument is influenced by a large number of variables – e.g., cultural, structural, and financial. Following data format unification and validation, each sample in the dataset contained 90 different fields of information, which was subsequently reduced to the following:

1. Preventive measures taken following risk assessment
2. Number of patients with pressure injuries
3. Categories (I-IV) of all pressure injuries of all patients
4. Physical location of the pressure injury(s) on the patient’s body.

### 4.4 Data analysis

The analysis was performed using MATLAB v2021b. Due to the large number of fields in the dataset, a custom graphical user interface for data exploration and live visualization was developed specifically for this study. The data were pre-processed and cleaned to compensate for different spellings or spelling mistakes in the record entries. Following this, the absolute number of patients assessed was extracted for each point prevalence measurement, as well as the assessment results and preventive measures taken. The percentage of patients who were diagnosed with at least one pressure injury among all assessed patients was extracted for each point prevalence measurement to allow for a normalized comparison among point prevalence measurements. Similarly, the number of respective preventive measures taken and incidence locations were normalized by the number of assessed patients and are reported in percent below. As the dataset contained all risk assessments performed during the point prevalence measurements, rather than representative samples, the normalized comparisons were not based on statistical hypotheses.

### 4.5 Ethical considerations

The head of the development strategy department at the hospital approved the study. It was conducted in accordance with the Declaration of Helsinki (WMA, 2013), as well as the national and local guidelines for research (CODEX, 2023). Research ethics committee approval was not required, there was no processing of sensitive personal information, and the study posed no physical or mental harm to the participants. Patients were provided with both verbal and written information about the point prevalence measurement by a member of the team (RNs or assistant nurses). Verbal consent was obtained at the time of the data collection during the point prevalence day to participate; if necessary, relatives were consulted. Participation was entirely voluntary, and all data were kept confidential.

## 5 Results

**Fig. 1:**
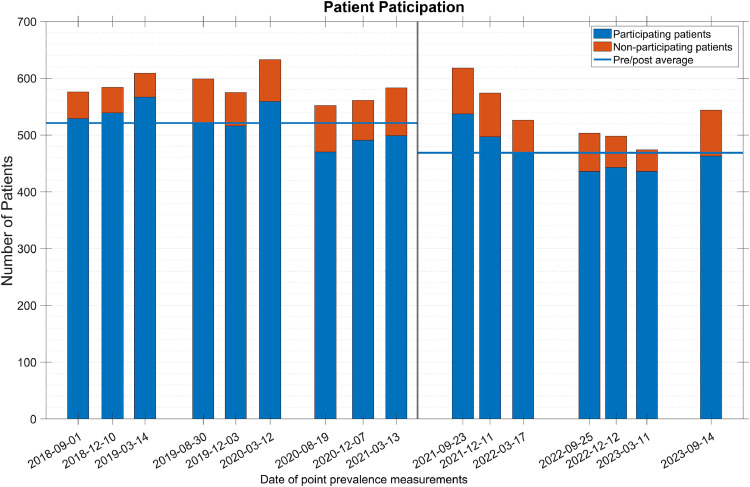
Total number of records from each point prevalence measurements with the number of patients declining to be risk assessed indicated by red bars, blue bars indicate participating patients, and pre- and post-introduction of PURPOSE T averages visualized by horizontal blue lines. The introduction of PURPOSE T is highlighted by a vertical grey line.

In total, the records of 9009 patients were included over 5 years. All departments, except psychiatry and the children’s department, participated. As the records contain only two sexes, the split was 52.9 % men and 47.1 % women, with 65.2 % of all participating patients 65 years or older. For an overview of the point prevalence measurement-specific counts, see Fig. 1.

### 5.1 Prevention interventions

**Fig. 2:**
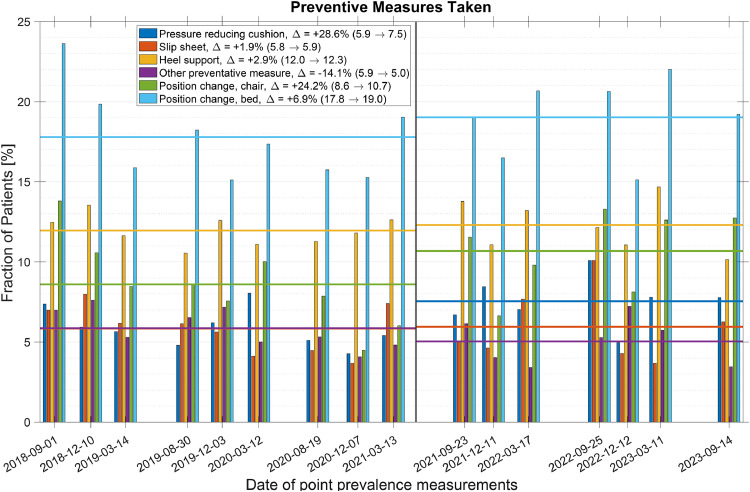
Preventative measures implemented at each point prevalence measurement(s) (vertical bars) and the pre- and post-PURPOSE T introduction averages of each measure (horizontal lines). The introduction is indicated by a single vertical grey line. Additionally, the changes in averages are in the legend indicated with a Δ, and, pre- and post-values are provided in the parentheses.

As seen in Fig. 2 there was an increase in all well-defined preventive measures. We observed a more substantial increase for some measures, such as pressure reducing chair cushions and position adjustments in chairs.

### 5.2 Pressure injury prevalence and category

**Fig. 3:**
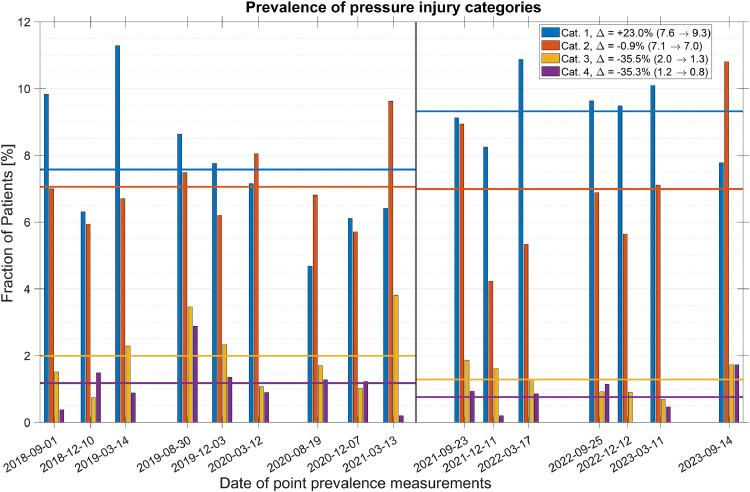
Categorized pressure injury prevalence (vertical bars) and their averages pre- and post- PURPOSE T introduction (horizontal lines). Additionally, the changes in averages are in the legend indicated with a Δ, and pre- and post-values are provided in the parentheses.

**Fig. 4:**
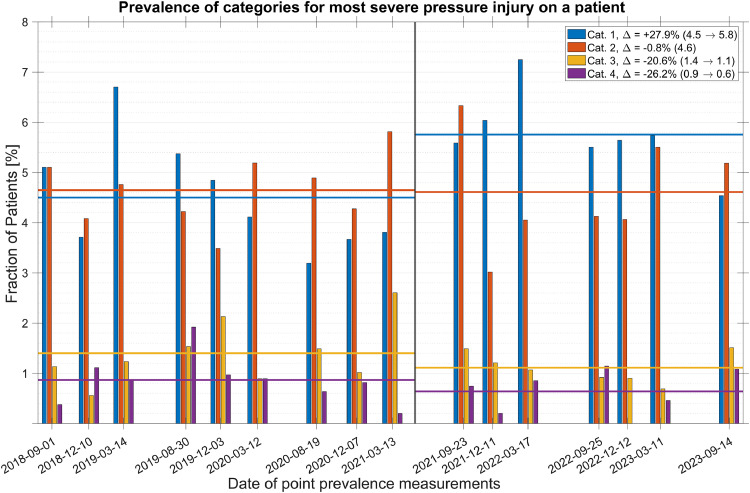
Most severe category of pressure injury found on patients with at least one pressure injury. Additionally, the changes in averages are in the legend indicated with a Δ, and pre- and post-values are provided in the parentheses.

The total prevalence of diagnosed pressure injuries over all point prevalence measurements was found to be 10.8 %. As seen in Fig. 3, the prevalence of pressure injuries was relatively consistent throughout the point prevalence measurements, with the average over the assessed time period before the introduction of PURPOSE T being 10.6 % and after, 11.1 %. However, following the introduction of PURPOSE T, there was a drastic shift in the categories of diagnosed pressure injuries. In the figure, this shift is highlighted by the pre- and post-averages of the different categories, with the least severe category increasing by almost a quarter and the two most severe ones decreased by more than a third each. Fig. 3 gives a clear view of the increase in early detection of pressure injuries, showing that the fraction of patients whose most severe pressure injury was of category I increased by almost 28 %. Regarding the distribution of location of pressure injuries on the patients’ bodies, it varied to some degree from measurement to measurement; however, over the time periods before and after the introduction of PURPOSE T, the incidence stayed nearly constant, indicating that the increase in early detection following PURPOSE T was not specific to any particular part of a patient’s body (Fig. 4).

## 6 Discussion

PURPOSE T is an evidence-based pressure injury risk assessment instrument that plays a vital role in enhancing patient safety, which is one of the cornerstones of high-quality healthcare systems. Evidence-based practice, as noted by Falk et al. (2023), is a key approach to improving patient safety from the nursing perspective.

The introduction of PURPOSE T led to an increased detection of certain risks and a subsequent modification in the implementation of preventive interventions, particularly in terms of timing and frequency. The use of the tool was not directly associated with a change in prevalence but rather with a shift in preventive practices. Notably, PURPOSE T influenced the distribution of pressure injury diagnoses, particularly in the early detection and management of these injuries. The data indicate a significant change in the prevalence of different pressure injury categories before and after implementation. Specifically, category I pressure injuries increased by nearly 25 %, while the most severe categories (III and IV) decreased by over 35 %. Although this is the first study evaluating the widespread implementation of PURPOSE T and no direct comparisons can be made with other studies, a 10-year follow-up in Sweden using different pressure injury risk assessment instruments (Modified Norton Scale and the Risk Assessment Pressure injury Scale) showed a decrease in pressure injury prevalence from 17.0 % to 13.9 %, without specifying changes among categories (Källman et al., 2022). This, in combination with the dramatic shift in pressure injury categories we observed, suggests that PURPOSE T contributed to an increase of preventive interventions and detecting pressure injuries early and thus to reducing the number of severe cases.

This interpretation of the results is supported by the clear changes in which preventative measures were used. As seen, pressure injuries of category I were identified at an early stage and, therefore, preventive measures, such as *sitting preventions* (chair cushion and change position in chair), were enabled, which increased by about a quarter, rather than the bedridden alternatives. These results are in line with previous studies that have shown that using a pressure injury risk assessment instrument increased the use of preventative interventions (Hödl et al., 2019; Fulbrook et al., 2023).

While the vast dataset used in this study could be employed in further statistical analysis, we have chosen to present primarily the preventive interventions and prevalence results that can be connected to the pressure injury risk assessment instrument. By doing so, it could be argued that the scope of the study was significantly limited. But, by using this approach, we expect the results and conclusions could be transferable to a wider setting.

One such conclusion that follows is that, when compared to the Modified Norton Scale, PURPOSE T did not seem to target any specific physical part of a patient’s body. The marked improvement in preventive interventions and in early detection was a holistic one, indicating that the approach of PURPOSE T did indeed improve upon previous pressure injury risk assessment instruments, rather than just tweak them.

As this is the first study of its kind, relating the results to previous work is not possible. Therefore, it is important to be restrained when drawing conclusions from the results. An uncontroversial one, however, is that with the improved preventive interventions and early detection rate following the introduction of PURPOSE T, the focus of pressure injury prevention interventions shifted towards mobilising patients in chairs instead of keeping them in beds. This shift, along with the reduction in prevalence of severe pressure injury categories, reduced the amount of pain experienced by patients and freed up time for healthcare staff.

## 7 Limitations

This study has several limitations. First, not all wards at the hospital participated in the point prevalence measurements, which may have influenced the results. However, the sample size did not vary significantly over the years, and the decrease in the number of participating patients from 2018 to 2023 could also be attributed to the reduction in occupied hospital beds that occurred during the time period. Comparing outcomes across different care settings would have been insightful; however, departmental reorganizations during this 5-year study period made such comparisons difficult.

From 2020 to 2022, the hospital was under significant strain due to the COVID-19 pandemic, and it is unclear how this may have affected the results due to the high pressure on patient flow and shortage of personnel. Therefore, conclusions based on data from those years should be drawn cautiously. Nevertheless, the dataset spans 5 years; i.e., a time span beyond the pandemic period.

Point prevalence measures are valuable but may not be as reliable as individual record reviews. This limitation arises because the point prevalence measurement process involves multiple steps: first, a nurse performs the risk assessment and records the results on paper. These results are then catalogued, revisited, and entered into a database. In contrast, during a *regular* risk assessment, there is only one step from the risk assessment to the patient’s record, without the intermediary step of recording results on paper.

## 8 Conclusions

We have shed light on the prevention and prevalence of pressure injuries at a hospital in Sweden following the widespread implementation of PURPOSE T. We have revealed a change in the preventive procedures, and this has led to a notable shift in pressure injury categories, with a decrease in categories II-IV and an increase in category I injuries after the hospital-wide implementation of PURPOSE T. The shift towards lower category pressure injuries may have led to reduced patient suffering, lower treatment costs, and shorter hospital stays, but further research is needed.

## Funding sources

This research received no specific grant from any funding agency in the public, commercial or not-for-profit sectors.

## Data availability

Data available on request from the authors.

## CRediT authorship contribution statement

**Ragnar Seton:** Writing – review & editing, Visualization, Software, Methodology, Formal analysis, Data curation. **Elisabeth Wetzer:** Writing – review & editing, Visualization. **Lisa Hultin:** Writing – review & editing, Writing – original draft, Methodology, Formal analysis, Conceptualization.

## Declaration of competing interest

The authors declare the following financial interests/personal relationships which may be considered as potential competing interests:

Lisa Hultin reports was provided by Uppsala University Department of Public Health and Caring Sciences. If there are other authors, they declare that they have no known competing financial interests or personal relationships that could have appeared to influence the work reported in this paper.
